# Genome-wide association study of height-adjusted BMI in childhood identifies functional variant in *ADCY3*

**DOI:** 10.1002/oby.20840

**Published:** 2014-07-21

**Authors:** Evangelia Stergiakouli, Romy Gaillard, Jeremy M Tavaré, Nina Balthasar, Ruth J Loos, Hendrik R Taal, David M Evans, Fernando Rivadeneira, Beate St Pourcain, André G Uitterlinden, John P Kemp, Albert Hofman, Susan M Ring, Tim J Cole, Vincent WV Jaddoe, George Davey Smith, Nicholas J Timpson

**Affiliations:** 1 MRC Integrative Epidemiology Unit at the University of BristolBristol, UK; 2 The Generation R Study Group, Erasmus Medical CenterRotterdam, The Netherlands; 3 Department of Epidemiology, Erasmus Medical CenterRotterdam, The Netherlands; 4 Department of Paediatrics, Erasmus Medical CenterRotterdam, The Netherlands; 5 School of Biochemistry, University of BristolBristol, UK; 6 School of Physiology and Pharmacology, University of BristolBristol, UK; 7 The Charles Bronfman Institute of Personalize Medicine, The Mindich Child Health and Development, The Icahn School of Medicine at Mount SinaiNew York, USA; 8 University of Queensland Diamantina Institute, Translational Research InstituteBrisbane, Queensland, Australia; 9 Department of Internal Medicine, Erasmus Medical CenterRotterdam, The Netherlands; 10 School of Oral and Dental Sciences, University of BristolBristol, UK; 11 School of Experimental Psychology, University of BristolBristol, UK; 12 Avon Longitudinal Study of Parents and Children (ALSPAC), School of Social and Community Medicine, University of BristolBristol, UK; 13 Population, Policy and Practice Programme, UCL Institute of Child HealthLondon, UK

## Abstract

**Objective:**

Genome-wide association studies (GWAS) of BMI are mostly undertaken under the assumption that “kg/m^2^” is an index of weight fully adjusted for height, but in general this is not true. The aim here was to assess the contribution of common genetic variation to a adjusted version of that phenotype which appropriately accounts for covariation in height in children.

**Methods:**

A GWAS of height-adjusted BMI (BMI[*x*] = weight/height^x^), calculated to be uncorrelated with height, in 5809 participants (mean age 9.9 years) from the Avon Longitudinal Study of Parents and Children (ALSPAC) was performed.

**Results:**

GWAS based on BMI[*x*] yielded marked differences in genomewide results profile. SNPs in *ADCY3* (adenylate cyclase 3) were associated at genome-wide significance level (rs11676272 (0.28 kg/m^3.1^ change per allele G (0.19, 0.38), *P* = 6 × 10^−9^). In contrast, they showed marginal evidence of association with conventional BMI [rs11676272 (0.25 kg/m^2^ (0.15, 0.35), *P* = 6 × 10^−7^)]. Results were replicated in an independent sample, the Generation R study.

**Conclusions:**

Analysis of BMI[*x*] showed differences to that of conventional BMI. The association signal at *ADCY3* appeared to be driven by a missense variant and it was strongly correlated with expression of this gene. Our work highlights the importance of well understood phenotype use (and the danger of convention) in characterising genetic contributions to complex traits.

## Introduction

BMI (weight(kg)/height(m^2^)) has become a uniformly used measure of weight given height despite being defined in the 19^th^ century based only on population specific knowledge at the time 1. As an index of weight for height it ought to be uncorrelated with height, but in practice it is not. This complicates its biological interpretation as the correlation between BMI and height varies across different age groups, body types and ethnicities 2,3. Different power terms [*x*] for height are required in the calculation of BMI in men and women and across different age groups and ethnicities to achieve maximum correlation with total body fat measured by Dual-energy X-ray absorptiometry (DXA) and minimum correlation with height 4. In addition, BMI is considered a measure of adiposity although it is particularly inaccurate for measuring adiposity in individuals with elevated lean body mass, such as athletes 5. BMI has been found previously not to be appropriate for children later in childhood, as the formula of weight(kg)/height(m^2^)) overestimates the BMI of tall or physically advanced children 6. Dividing BMI by the appropriate power of height as a function of the child's age was suggested as the best way to assess adiposity in children 6. However, the genetic profiles of BMI versus suggested alternative measurements of adiposity in children have not been assessed.

Phenotypic refinement is important in the undertaking of informative and well powered GWAS. Not only can the use of more biologically proximal measurements reduce the level of noise associated with any given genetic association signal, but the redefinition of a routine measurement can yield marked differences in genetic profile. A clear example of this was seen in the publication of the now well-known association between variation at *FTO* and fat mass 7,8. Initially, *FTO* was discovered as a type 2 diabetes (T2D) locus, as reported in an association study for T2D in the absence of BMI matching in cases and controls 9. The combination of study design and phenotypic refinement allowed for the demonstration that *FTO* was exerting an indirect effect on T2D risk through its relationship with BMI 7,8. Concerning anthropometry, the assessment of genome-wide contributions of common variants to waist-to-hip ratio (WHR) and WHR adjusted for BMI are examples of association studies where relatively simple anthropometric measurements have been refined through either subtype or adjustment and have yielded novel genome-wide association profiles 10,11.

Although BMI was designed to assess weight independent of height, it remains correlated with height owing to its generalized derivation. This correlation changes throughout the life course and has the potential to complicate inference and reduce power in association studies. Targeting this well-known, but often ignored, limitation in BMI as a measure for fat mass we aimed to assess the contribution of common genetic variation to a height-adjusted version of that phenotype which appropriately accounts for covariation in height in children. Using data available from the Avon Longitudinal Study of Parents and Children (ALSPAC) study, we set out to undertake a genome-wide association study (GWAS) for a height-adjusted version of BMI using the appropriate power function for minimizing the correlation between BMI and height in the age group with the largest correlation between weight and height.

## Methods

### ALSPAC

ALSPAC is a prospective birth cohort which recruited pregnant women with expected delivery dates between April 1991 and December 1992 from Bristol UK. About 14,541 pregnant women were initially enrolled with 14,062 children born. Detailed information on health and development of children and their parents were collected from regular clinic visits and completion of questionnaires. A detailed description of the cohort has been published previously 12,13. Ethical approval was obtained from the ALSPAC Law and Ethics Committee and the Local Ethics Committees. Please note that the study website contains details of all the data that is available through a fully searchable data dictionary (http://www.bris.ac.uk/alspac/researchers/data-access/data-dictionary/).

A total of 9912 participants were genotyped using the Illumina HumanHap550 quad genome-wide SNP genotyping platform. Quality control assessment and imputation are described in Supporting Information. After quality control assessment and imputation the data set consisted of 8365 individuals 2,608,006 SNPs available for analysis.

### Generation R

The Generation R Study is a population-based prospective cohort study of pregnant women and their children from fetal life onwards in Rotterdam, The Netherlands 16,17. All children were born between April 2002 and January 2006, and currently followed until young adulthood. Of all eligible children in the study area, 61% were participating in the study at birth. Anthropometric data were collected in several developmental stages. Cord blood samples including DNA have been collected at birth. The current study used the first set of Generation R samples of Northern European Ancestry.

Samples were genotyped using Illumina Infinium II HumanHap610 Quad Arrays following standard manufacturer's protocols. Quality control assessment and imputation are described in Supporting Information. After quality control assessment and imputation 2729 children and 2,543,887 SNPs were included in the analyses.

### Phenotype calculation

For ALSPAC, we used measurements of height and weight at clinic visits when the children were nine years of age to calculate BMI. Height was measured to the last complete mm using the Harpenden Stadiometer. A total of 5809 unrelated children had both anthropometric and genetic data appropriate for our analysis. Their mean age was 9.9 years (SD 0.3), the mean BMI was 17.7 (SD 2.8), mean height was 139.5 cm (SD 6.3) and 50.5% were female. A Lunar prodigy narrow fan beam densitometer was used to perform a whole body DXA (Dual-energy X-ray absorptiometry) scan where bone content, lean and fat masses are measured 18,19. BMI was also measured as above in Generation R at regular intervals with the latest measurement used for this analysis [mean age = 6.1 years (SD 0.4)]. A total of 2089 unrelated children had both anthropometric and genetic data appropriate for our analysis with mean BMI 15.9 (SD 1.4), mean height 119.5 cm (SD 5.6).

In ALSPAC, we defined height-adjusted BMI as BMI[*x*] = weight(kg)/height(m)^*x*^). For each age group we calculated BMI[*x*] iteratively increasing the power [*x*] term by 0.1 each time. We then measured the correlation of each BMI[*x*] with height (within children of the same age group) based on Pearson's correlation coefficient. We selected the power term that resulted in the lowest correlation coefficient of BMI[*x*] with height for any given age. This approach yielded a value for BMI[*x*] which performed equivalently to adjusting log BMI for log height 6, but which reported the appropriate power term for each age group and thus the relative inefficiency of conventional BMI. In addition, we calculated zBMI, which is a commonly used measure in clinical practice, by standardising BMI by age and sex. Stata 12 was used for the calculations 20. In Generation R BMI was calculated as BMI = weight(kg)/height(m)^2^) and height was included as a covariate in the GWAS model.

### Statistical methods

Genomewide association analyses were performed using MACH2QTL V110 (14,15). To investigate if the association at the adenylate cyclase 3 (*ADCY3)* locus could be attributed to rs11676272, conditional analysis was performed by including the rs11676272 dose generated during imputation in the regression model and conducting regional single marker association analyses with BMI[3.1]. Age, sex, height, and rs11676272 dose were included as covariates in the model. Plots were generated using LocusZoom 21.

For the meta-analysis of BMI adjusted for height in ALSPAC and Generation R samples, SNPs that had a minor allele frequency <0.01 and an *r*^2^ imputation quality score <0.3 were excluded. Meta-analysis was performed using METAL software package 22. Sample size weighted analysis (based on *P*-values) was conducted. We used the “Heterogeneity” command in METAL to assess evidence of between study heterogeneity.

Body fat and lean mass were measured by whole body DXA in 5557 children during a clinic performed at age 9 as described above. Data were log transformed and then *z* scores were calculated for the transformed values assuming normality. Linear regressions were performed with age, sex, and height as covariates in the two models. In addition, bi-directional adjustment was performed with lean mass included as a covariate in the model for body fat and body fat included as a covariate in the model for lean mass. Association of rs11676272 with both body fat and lean mass would suggest that the height-adjusted BMI phenotype reflects changes in weight, while an association with body fat alone would be expected if it reflects fat mass specifically. Stata 12 was used for these calculations 20.

### Expression

RNA was extracted from lymphoblastoid cell line (LCLs) generated from 997 unrelated ALSPAC individuals of age 9, which were included in the GWAS, using an RNeasy extraction kit (Qiagen) and was amplified using the Illumina TotalPrep-96 RNA Amplification kit (Ambion). Expression was evaluated using Illumina HT-12 v3 BeadChip arrays. Each individual sample was run with two replicates. Expression data were normalized by quantile normalization between replicates and then by median normalization across individuals. For 949 ALSPAC individuals, both expression levels and imputed genome-wide SNP data were available. We used linear regression to investigate the association between rs11676272 and any transcript within 500 kb of this SNP.

Genevar, a database and Java application for the analysis and visualization of SNP-gene associations in eQTL studies 23, was used to test for evidence of *ADCY3* expression in public databases. *ADCY3* expression was analyzed in data from 856 healthy female twins of the Multiple Tissue Human Expression Resource (MuTHER) resource in both adipose and lymphoblastoid cell lines 24.

## Results

**Figure 1:**
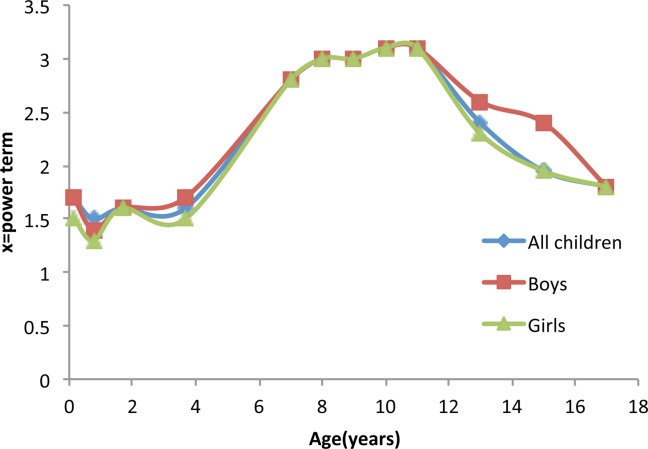
Power term [*x*] required for the least correlation of BMI[*x*] = weight/(height)x with height. This is reflected by the correlation coefficient across different age groups in all children from the Avon Longitudinal Study of Parents and Children (ALSPAC) study and stratified by sex. [Color figure can be viewed in the online issue, which is available at wileyonlinelibrary.com.]

The height power *x* defining BMI[*x*] varied by age from infancy to 17 years (Figure 1). It was smallest at 12 months (*x* = 1.5) and largest at 8-12 years (*x* = 3.1), this latter period being the time when BMI[*x*] and BMI (with *x* = 2) were most different. This is also illustrated by the correlation of standard BMI with height which was maximum during infancy and remained high until 13 years when puberty commences (Supporting Information Figure S1**).** zBMI also suffers the same problem of correlation with height as BMI (Supporting Information Figure S2)**.**

**Figure 2:**
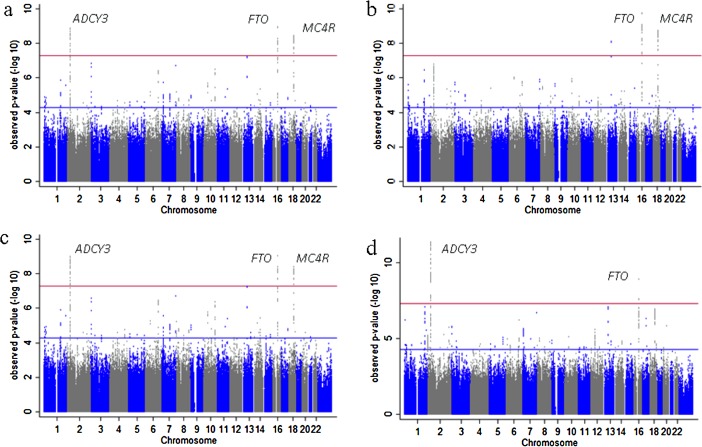
Manhattan plots of the results from genome-wide association studies (GWAS) on BMI. (a) ALSPAC GWAS on BMI[3.1] adjusted for sex and age, (b) ALSPAC GWAS on BMI adjusted for sex and age, (c) ALSPAC GWAS on BMI adjusted for sex, age, and height, and (d) meta-analysis of ALSPAC and Generation R GWAS on BMI adjusted for sex, age, and height. [Avon Longitudinal Study of Parents and Children (ALSPAC)].

Selecting 8-12 years as the age which both maximises sample size and exploit the difference between BMI[*x*] and BMI, we performed a GWAS of BMI[*x*] with *x* = 3.1, adjusted for age and sex, where *x* was estimated from 5809 nonrelated participants seen at the age 9 clinic visits of the ALSPAC study (mean age = 9.9 years). The strongest signal of association between genetic variation and BMI[3.1] was rs1558902 at *FTO* (0.21 kg/m^3.1^ change per allele A (0.13, 0.27), *P* = 9 × 10^−10^, minor allele frequency (MAF) *A* = 0.4) (Figure 2a). However, a similar effect was found for rs11676272 (0.20 kg/m^3.1^ change per allele *G* (0.13, 0.27), *P* = 4 × 10^−9^, MAF *G* = 0.48), a nonsynonymous SNP located in the first exon of *ADCY3* resulting in a serine-to-proline substitution in the second transmembrane helix of the expressed protein.

**Figure 3:**
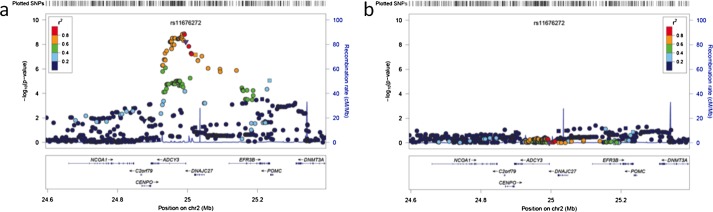
Regional plots in ADCY3(adenylate cyclase 3) locus showing that the association at the locus is attributed to rs11676272. (a) BMI adjusted for height, age and sex in ALSPAC, (b) BMI adjusted for height, age and sex conditioning on rs11676272 in ALSPAC. Each circle represents a SNP plotted by its position on the chromosome against its association (−log10 *P*). The SNPs are colored according to their linkage disequilibrium (LD) with rs11676272 using pairwise *r*^2^ values from HapMap CEU samples. The diagram in light blue represents the LD structure in the region. Data for gene structure from UCSC Genome Browser are shown below the diagram. Plots were generated using LocusZoom 22 [Avon Longitudinal Study of Parents and Children (ALSPAC)].

For variation at SNPs across the *ADCY3* locus and conventional BMI there was not genome-wide significant evidence for association (Figure 2b) [rs11676272 (0.25 kg/m^2^ (0.15, 0.35), *P* = 6 × 10^−7^)]. zBMI (BMI standardised for age) was not associated with rs11676272 [0.022 change in zBMI per allele *G* (−0.01 to 0.06), *P* = 0.224)]. In contrast, the association of rs1558902 in *FTO* remained genome-wide significant [0.33 kg/m^2^ (0.23, 0.43), *P* = 1 × 10^−10^]. Evidence for association at the *ADCY3* locus and rs11676272 [0.28 kg/m^2^ (0.19, 0.38), *P* = 6 × 10^−9^] remained genome-wide significant, as expected, when performing a GWAS on conventional BMI adjusted for height (Figure 2c). Furthermore, in sensitivity analyses performing regional single marker association analyses with BMI adjusted for height conditional on rs11676272 dose, all evidence for association across the *ADCY3* region was lost (Figure 3).

There was consistent evidence of association for rs11676272 with BMI adjusted for height [0.14 kg/m^2^ (0.06, 0.22), *P* = 0.0001] in 2089 nonrelated children from Generation R (mean age = 6.1 years). This time point in Generation R children was selected as it was the latest age available and the closest to children of age nine in ALSPAC. A meta-analysis of BMI adjusted for height in ALSPAC and Generation R samples provided additional evidence for association between rs11676272 and BMI adjusted for height (Figure 2d). Again, the phenotypic association across this region of chromosome 2 was ablated when adjusted for rs11676272 dose.

Unlike the age-dependent association of *FTO* and BMI 25 the association of *ADCY3* with BMI[*x*] was consistent across age. The strength of association of rs11676272 with BMI[*x*] at age 7 (*x* = 2.8) was similar to that at age 9 [0.17 kg/m^2.8^ (0.11, 0.23), *P* = 9 × 10^−9^] while as expected 25, the association of rs9939609 in *FTO* was weaker in younger children [age 7: 0.09 kg/m^2.8^ (0.03, 0.15), *P* = 0.002].

rs11676272 was strongly associated with total fat mass with height in the model [0.11 kg (0.08, 0.14), *P* = 7 × 10^−12^], but adding lean mass to the model made no difference [0.1 kg (0.07, 0.13), *P* = 6 × 10^−11^]. Furthermore, lean mass was only weakly associated with variation at rs1167612 [0.02 kg (0.001, 0.041), *P* = 0.04], disappearing completely when adjusted for body fat (0.00 kg (−0.02, 0.02), *P* = 0.8). The correlation of BMI[3.1] with fat mass (*r* = 0.84) was far stronger than with lean mass (r = 0.31) (Supporting Information Figure S3).

In contrast to the association of *ADCY3* with BMI[3.1] and total fat mass, rs11676272 was not associated with height in ALSPAC [−0.19 cm (−0.41, 0.03), *P* = 0.1] while the SNP in the *ADCY3* region best associated with height was rs2918630 [0.30 cm (0.08, 0.53), *P* = 0.008].

**Figure 4:**
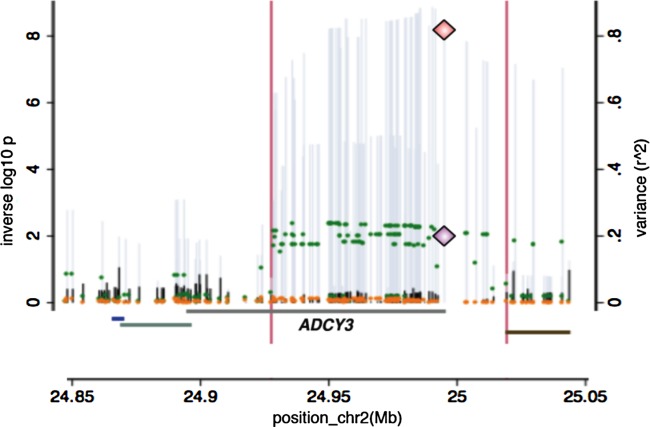
Genetic and expression data on the ADCY3 (adenylate cyclase 3) locus. Overlay of the GWAS on BMI adjusted for height results (left axis and grey spikes), the GWAS on BMI adjusted for height conditioning on rs11676272 dose (left axis and black spikes), the association of the expression of ADCY3 by the same variants in the region (expressed as *r*^2^ on the right axis and green dots) and the residual variation in expression after regression on rs11676272 (orange dots). The diamonds indicate the position of rs11676272 p values for the test for association with BMI adjusted for height (top diamond) and gene expression results expressed as *r*^2^ (bottom diamond).

Analysis of *ADCY3* expression in lymphoblastoid cell lines showed a strong association between the rare (adiposity increasing) variant *G* at rs11676272 and reduced levels of *ADCY3* expression [−0.65 SD per allele (−0.74, −0.57), *P* = 1 × 10^−53^]. Conditioning on rs11676272 dose abolished this association, reducing the variance in expression levels explained by each SNP from *r*^2^ = 0.21 to *r*^2^ < 0.01 (Figure 4). In MuTHER study data, expression analyses showed strong evidence of *ADCY3* expression in both adipose (Supporting Information Figure S4 and Table S1) and lymphoblastoid cell lines (Supporting Information Figure S5 and Table S2) 24 with the rare variant *G* at rs11676272 associated with reduced levels of expression in adipose tissue [−0.1 SD per allele (−0.08, −0.13), *P* = 4 × 10^−8^).

## Discussion

BMI is a widely accepted though imperfect index of weight for height, which is complicated by differential performance in certain body types and age groups. We used GWAS to investigate the contribution of common variants to an index of weight (and equivalently BMI) uncorrelated with height in children of age 9 from the ALSPAC study. A missense variant, rs11676272, in *ADCY3* showed evidence of genome-wide significance only when height was adjusted for, and this result was replicated in an independent sample of children from the Generation R study.

Our results using BMI[*x*] highlight the importance of the timing of puberty for height and weight measurements 26,27. During puberty the correlation between weight and height increases, so weight adjusted for height requires a larger height power, as seen in Figure 1 6. This arises because both weight and height are temporarily greater in earlier than later maturers, which stretches the joint distribution. BMI only partially adjusts weight for height, so it includes a residual height signal which means that BMI is also raised in early maturers. However, if weight is fully adjusted for height (i.e. using BMI[*x*]) the residual height signal is largely abolished, and BMI[*x*] is much closer to a measure of adiposity independent of the timing of puberty. The same limitation is also found in zBMI, which, although takes age and sex into account, is still correlated with height.

This is relevant because our previous work 27 found that the relation between BMI and *FTO* related at least in part to developmental age, i.e. that children with the obesity-variant had an earlier adiposity rebound, a predictor of later obesity. By using BMI[*x*] rather than BMI we have reduced (though not removed) the developmental age signal, which has allowed the adiposity signal to emerge via its association with the *ADCY3* locus. In this case, signals at loci such as *ADCY3* fall into a new category of signals for BMI in the absence of coincident correlations with height that may suggest that they are having a more basal adiposity effect and one which should be more consistent with time.

**Figure 5:**
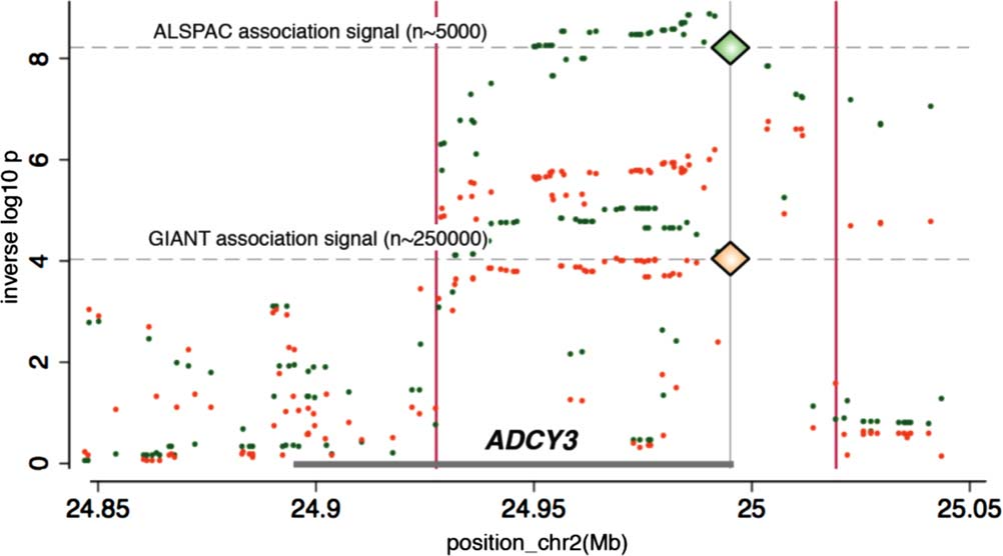
Regional plot for ADCY3 (adenylate cyclase 3) locus with ALSPAC and the Genetic Investigation of ANthropometric Traits (GIANT) meta-analysis data 11. Green circles represent SNPs and their association with BMI[3.1] in ALSPAC and orange circles represent SNPs and their association with BMI in the GIANT meta-analysis. rs11676272 is indicated by a diamond. [Avon Longitudinal Study of Parents and Children (ALSPAC)].

The *ADCY3* locus has been implicated in genetic studies of body weight and height before. SNPs in the *ADCY3* gene were associated with obesity in a sample of Swedish men with and without type 2 diabetes 28. This locus, though not this variant, was found to be associated with BMI in the GIANT (Genetic Investigation of ANthropometric Traits) consortium meta-analysis (rs10182181, *P* = 1.8 × 10^−7^, *r*^2^(with rs11676272)= 0.97) 29 and also in a meta-analysis of non- European populations (rs6545814, *P* = 1.35 × 10^−13^, *r*^2^ (with rs11676272)= 0.8) 30. Unlike the ∼5000 children that were used in our study, both of these studies had sample sizes exceeding 120,000 individuals. Furthermore, the signal reported at rs11676272 (i.e. a directly implicated functional variant) presented weak evidence (consistent with chance) in the basic large-scale meta-analyses for BMI undertaken in the GIANT investigation (rs11676272: *P* = 9.3 × 10^−5^) despite the size of the study (Figure 5).

**Figure 6:**
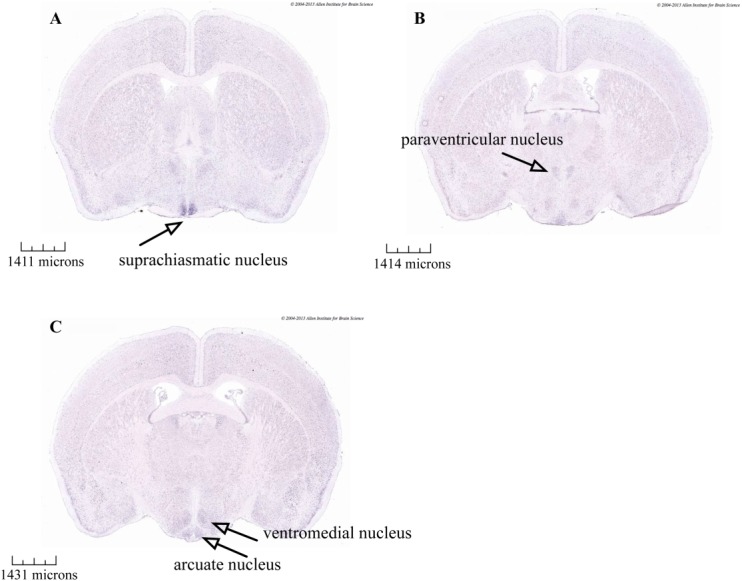
In situ hybridization data from the Allen Mouse Brain Atlas. Darker staining is seen in coronal sections of adult mouse brains indicating ADCY3 (adenylate cyclase 3) mRNA expression within the hypothalamus, including the (a) suprachiasmatic, (b) paraventricular, (c) ventromedial, and (d) arcuate nucleus. Data from Allen Mouse Brain Atlas 35 (http://mouse.brainmap.org/experiment/ivt?id=75&popup=true). [Color figure can be viewed in the online issue, which is available at wileyonlinelibrary.com.]

*ADCY3* codes for the membrane-associated enzyme adenyl cyclase 3, which catalyses the synthesis of cAMP from ATP 31. The ACDY3 protein sequence comprises two clusters of membrane spanning helices, called M_1_ and M_2_, which interact to bring together a large cytosolic intracellular loop with a region at the C-terminus to form a composite and competent catalytic domain 32. The serine-to-proline substitution coded by rs11676272 lies within the second transmembrane spanning alpha-helix of the M_1_ cluster. In addition to the observed association with reduced expression of *ADCY3*, we suggest that the proline substitution could disrupt the interaction between helix bundle M_1_ and M_2_ leading to a reduction in adenyl cyclase activity.

*In situ* hybridization data from the Allen Mouse Brain Atlas 33, which integrates extensive gene expression and neuroanatomical data, show *ADCY3* mRNA expression in the mouse brain within several nuclei of the hypothalamus, including the paraventricular, ventromedial, and arcuate nucleus (Figure 6) 33,34, regions that are involved in central regulation of energy homeostasis. *ADCY3* knockout mice exhibit age-dependent obesity, which was attributed to hyperphagia, low locomotor activity, and leptin insensitivity and demonstrated to be most likely because of hypothalamic cAMP reductions 35.

The strength of our study is in the availability of cross-sectional anthropometric data at multiple time points for a large number of children. It enabled us to identify that variation in *ADCY3* is associated with body fat rather than lean mass or height in the ALSPAC sample. Variation at *ADCY3* has been associated with height (rs4665736, *P* = 1.44 × 10^−13^ in the GIANT meta-analysis for height) 36. However, in children of age 9 from the ALSPAC study rs11676272 was not associated with height [−0.189cm change per allele *G* (−0.041,0.034), *P* = 0.098].

## Conclusion

We have identified a functional polymorphism that is associated with fat mass in childhood with an effect size comparable to common variation at the *FTO* locus, however, only when height is correctly taken into account. In the general context of several limitations of BMI (i.e. age, ethnicity, body composition), our work highlights the potential impact to analytical precision/power associated with naïve use of BMI. In this investigation of height-adjusted BMI we have been able to show, in addition to limited available evidence, strong association with functional variation at *ADCY3*. Currently, it is not clear how this polymorphism can lead to the increased body fat observed, though effects could be directly on adipose tissue, triglyceride metabolism and/or the result of a centrally-mediated hyperphagic response.
