# Supplementary material for: Genome-wide association study of height-adjusted BMI in childhood identifies functional variant in ADCY3
Source: Obesity (Silver Spring). 2014 Jul 21;22(10):2252–9. doi: 10.1002/oby.20840 (PMC4265207; doi:10.1002/oby.20840)
Supplement: Supplementary file 1 [file oby0022-2252-SD1.docx]

**Genome-wide association study of height-adjusted BMI in childhood identifies functional variant in *ADCY3***

Evangelia Stergiakouli^1^, Romy Gaillard^2,3,4^, Jeremy M. Tavaré^5^, Nina Balthasar^6^, Ruth J. Loos^7^, Hendrik R. Taal^2,3,4^, David M. Evans^1,8^, Fernando Rivadeneira^3,9^, Beate St Pourcain^1,10,11^, André G. Uitterlinden^3,9^, John P. Kemp^1,8,12^, Albert Hofman^3^, Susan M. Ring^12^, Tim J. Cole^13^, Vincent W.V. Jaddoe^2,3,4^, George Davey Smith^1^, Nicholas J. Timpson^1^

^1^MRC Integrative Epidemiology Unit at the University of Bristol, Bristol, UK. Correspondence: Nicholas J. Timpson ([n.j.timpson@bristol.ac.uk](mailto:n.j.timpson@bristol.ac.uk)) ^2^The Generation R Study Group, Erasmus Medical Center, Rotterdam, The Netherlands ^3^Department of Epidemiology, Erasmus Medical Center, Rotterdam, the Netherlands ^4^Department of Paediatrics, Erasmus Medical Center, Rotterdam, the Netherlands ^5^School of Biochemistry, University of Bristol, Bristol, UK ^6^School of Physiology and Pharmacology, University of Bristol, Bristol, UK ^7^The Charles Bronfman Institute of Personalize Medicine, The Mindich Child Health and Development, The Icahn School of Medicine at Mount Sinai, New York, USA ^8^University of Queensland Diamantina Institute, Translational Research Institute, Brisbane, Queensland, Australia ^9^Department of Internal Medicine, Erasmus Medical Center, Rotterdam, The Netherlands ^10^School of Oral and Dental Sciences, University of Bristol, Lower Maudlin Street, Bristol BS1 2LY, UK ^11^School of Experimental Psychology, University of Bristol, Bristol, UK ^12^Avon Longitudinal Study of Parents and Children (ALSPAC), School of Social and Community Medicine, University of Bristol, Bristol, UK ^13^MRC Centre of Epidemiology for Child Health, UCL Institute of Child Health, London, UK

Correspondence: Dr Nicholas Timpson, MRC Integrative Epidemiology Unit at the University of Bristol, Oakfield House, Oakfield Grove, BS8 2BN, Bristol, UK. Tel: +44 1173310131; Fax: +44 1173310080; Email: [n.j.timpson@bristol.ac.uk](mailto:N.J.Timpson@bristol.ac.uk)

**SUPPLEMENTARY INFORMATION**

**Methods**

**ALSPAC GWAS**

A total of 9,912 participants were genotyped using the Illumina HumanHap550 quad genome-wide SNP genotyping platform by Sample Logistics and Genotyping Facilities at the Wellcome Trust Sanger Institute and LabCorp (Laboratory Corportation of America) supported by 23andMe. Individuals were excluded from further analysis on the basis of having incorrect sex assignments; extreme heterozygosity (<0.320 and >0.345 for the Sanger data and <0.310 and >0.330 for the LabCorp data); high levels of individual missingness (>3%); evidence of cryptic relatedness (>10% IBD) and being of non-European ancestry (as detected by a multidimensional scaling analysis seeded with HapMap 2 individuals). EIGENSTRAT analysis revealed no additional obvious population stratification and genome-wide analyses with other phenotypes indicate a low lambda. The resulting data set consisted of 8,365 individuals. SNPs with a minor allele frequency of <1% and call rate of <95% were removed. Only SNPs which passed an exact test of Hardy–Weinberg equilibrium (p >5 × 10^-7^) were considered for analysis. Known autosomal variants were imputed with MACH 1.0.16 Markov Chain Haplotyping software ([1](#_ENREF_1), [2](#_ENREF_2)), using CEPH individuals from phase 2 of the HapMap project (HG18) as a reference set (release 22). For the X chromosomal variants, imputation was performed using MiniMac (v4.43) ([3](#_ENREF_3)) and CEPH individuals from phase 3 of the HapMap project (HG18) were used as the reference set. After imputation, SNPs with a minor allele frequency <0.01 and an r^2^ imputation quality score <0.3 were excluded and this resulted in 2,608,006 SNPs available for analysis. Association analyses were performed using MACH2QTL V110 ([1](#_ENREF_1), [2](#_ENREF_2)).

**Generation R GWAS**

Samples were genotyped using Illumina Infinium II HumanHap610 Quad Arrays following standard manufacturer's protocols. Intensity files were analyzed using the Beadstudio Genotyping Module software v.3.2.32 and genotype calling based on default cluster files. Single marker association analyses with BMI were performed using an additive genetic model implemented in MACH2QTL ([2](#_ENREF_2)). Age, sex and height were included as covariates in the model according to the analyses. Any sample displaying call rates below 97.5%, excess of autosomal heterozygosity (F<mean-4SD) and mismatch between called and phenotypic sex were excluded. In addition, individuals identified as genetic outliers by the IBS clustering analysis (> 3 standard deviations away from the HapMap CEU population mean) and one of 2 pairs of identical twins (IBD probabilities =1) were excluded from the analysis. After quality control (QC) 2,729 children were included in the analyses. Genotypes were imputed for all polymorphic SNPs from phased haplotypes in autosomal chromosomes of the HapMap CEU Phase II panel (release 22, build 36) oriented to the positive (forward) strand. Genotyped SNPs with minor allele frequency < 0.01, SNP Call Rate < 0.98 and HWE P-value < 1x10^-6^ were filtered. After marker pruning 503,248 SNPs were used for imputation (MACH v 1.0.16) of 2,543,887 SNPs. Association analysis for directly genotyped data were carried out in PLINK implemented on BCSNPmax and for imputed data were ran using MACH2DAT implemented in the GRIMP27 user interface platform. The study protocol was approved by the Medical Ethical Committee of the Erasmus Medical Centre, Rotterdam (MEC 217.595/2002/20). Written informed consent was obtained from all participants.

The graph in **Figure S1** shows the correlation of BMI with height by age. The correlation is highest in early life and remains high until 11 years, after which it falls close to zero.

**
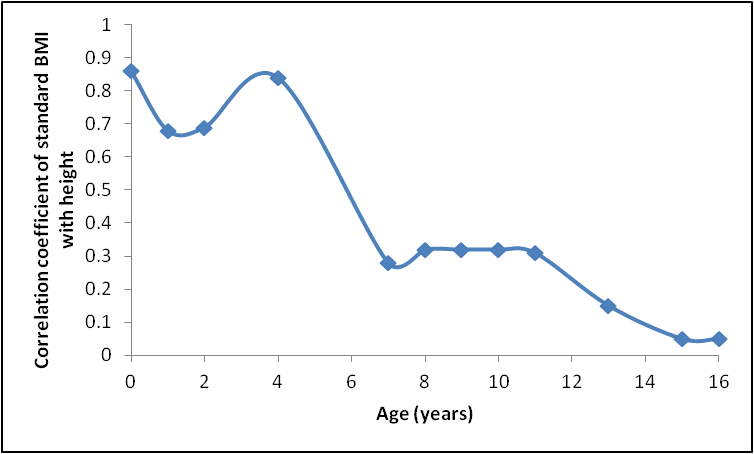
**

**Figure S1. Correlations of BMI and height by age in children from the Avon Longitudinal Study of Parents and Children (ALSPAC). The correlation coefficient was calculated for each age group separately.**

The graph in **Figure S2** shows the correlation of zBMI (BMI standardised by age) with height by age. The correlation remains high from age nine until age 13.


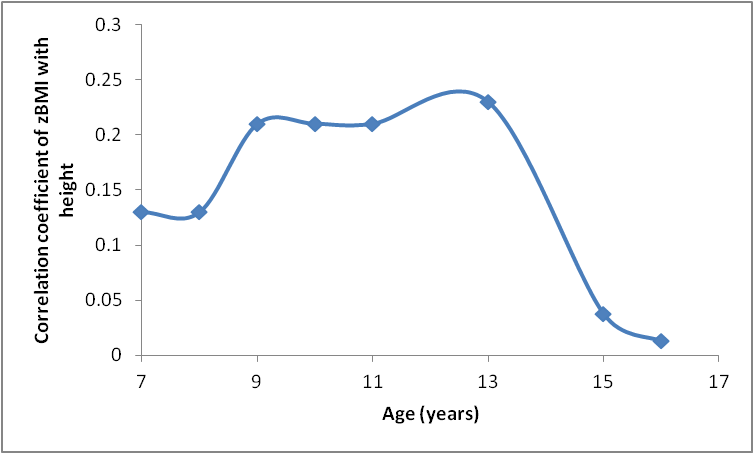


**Figure S2. Correlation coefficient of zBMI, standardised for age and sex, with height across different age groups in all children from the Avon Longitudinal Study of Parents and Children (ALSPAC). The correlation coefficient of BMI with height was calculated for each age group separately.**

The scatter plots in **Figure S3** show the correlation of BMI[*x*] at different ages with lean mass and body fat. The correlation of BMI[*x*] with body fat was stronger (Pearson’s correlation coefficient: 0.84) than the one of BMI[*x*] with lean mass (Pearson’s correlation coefficient: 0.31), strengthening our notion that BMI[*x*] is a measure of total body fat.

Figure S3. Scatter plots of lean mass and body fat against BMI_ht_

**Association of *ADCY3* with expression data from public databases**

Genevar, a database and Java application for the analysis and visualization of SNP-gene associations in eQTL studies ([4](#_ENREF_4)), was used to test for evidence of *ADCY3* expression in public databases. Analysis of *ADCY3* expression in data from 856 healthy female twins of the MuTHER resource showed strong evidence of *ADCY3* expression in both adipose **(Figure S4 and Table S1)** and lymphoblastoid cell lines **(Figure S5 and Table S2)** ([5](#_ENREF_5)).


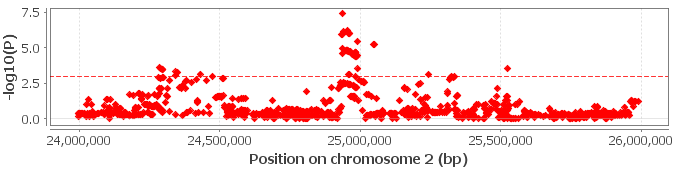


Figure S4. Regional plot for ADCY3 locus showing expression in adipose cell lines from 856 healthy female twins of the MuTHER resource. Each diamond represents a SNP plotted y its position on the chromosome against its association (-log10 P). Plots were created using Genevar ([4](#_ENREF_4)).

Table S1. Results of analysis of expression in adipose cell lines at the *ADCY3* locus using data from 856 healthy female twins of the MuTHER resource ([5](#_ENREF_5)). Results were created using Genevar ([4](#_ENREF_4)). Only the first 20 SNPs are shown.

| SNP ID | SNP Position | A1 | INFO | Freq1 | beta_SNP | sebeta_SNP | chi | P |
| --- | --- | --- | --- | --- | --- | --- | --- | --- |
| rs7576788 | 24936814 | T | 0.971 | 0.55 | -0.0871 | 0.0158 | 30.31 | 3.68E-08 |
| rs2033655 | 24954596 | G | 1 | 0.56 | -0.0764 | 0.0153 | 24.903 | 6.03E-07 |
| rs10865315 | 24954242 | C | 0.998 | 0.56 | -0.076 | 0.0153 | 24.632 | 6.94E-07 |
| rs1529897 | 24940331 | T | 0.995 | 0.562 | -0.0761 | 0.0154 | 24.52 | 7.35E-07 |
| rs7591460 | 24957471 | C | 0.994 | 0.44 | 0.0754 | 0.0154 | 24.136 | 8.98E-07 |
| rs11686663 | 24961263 | T | 0.992 | 0.439 | 0.0754 | 0.0154 | 24.025 | 9.51E-07 |
| rs1865689 | 24961701 | T | 0.992 | 0.561 | -0.0753 | 0.0154 | 24.022 | 9.53E-07 |
| rs1541984 | 24935918 | G | 0.999 | 0.563 | -0.0747 | 0.0153 | 23.739 | 1.10E-06 |
| rs11675457 | 24933274 | T | 0.998 | 0.437 | 0.0746 | 0.0153 | 23.651 | 1.16E-06 |
| rs2384061 | 24989124 | G | 0.999 | 0.562 | -0.0708 | 0.0153 | 21.516 | 3.51E-06 |
| rs6749646 | 25047502 | T | 0.981 | 0.224 | 0.0833 | 0.0184 | 20.523 | 5.89E-06 |
| rs13388020 | 25049770 | G | 0.981 | 0.776 | -0.0832 | 0.0184 | 20.5 | 5.96E-06 |
| rs11687089 | 24936430 | T | 0.994 | 0.572 | -0.0674 | 0.0153 | 19.394 | 1.06E-05 |
| rs2033656 | 24954406 | G | 0.997 | 0.458 | 0.0669 | 0.0155 | 18.726 | 1.51E-05 |
| rs2384059 | 24953842 | T | 0.996 | 0.457 | 0.0669 | 0.0155 | 18.724 | 1.51E-05 |
| rs6545776 | 24952861 | C | 0.993 | 0.457 | 0.067 | 0.0155 | 18.717 | 1.52E-05 |
| rs2384058 | 24953832 | G | 0.996 | 0.457 | 0.0669 | 0.0155 | 18.713 | 1.52E-05 |
| rs11892869 | 24950196 | T | 0.992 | 0.457 | 0.0669 | 0.0155 | 18.704 | 1.53E-05 |
| rs7567997 | 24950456 | T | 0.992 | 0.543 | -0.0669 | 0.0155 | 18.702 | 1.53E-05 |
| rs7580081 | 24950576 | G | 0.992 | 0.543 | -0.0669 | 0.0155 | 18.702 | 1.53E-05 |


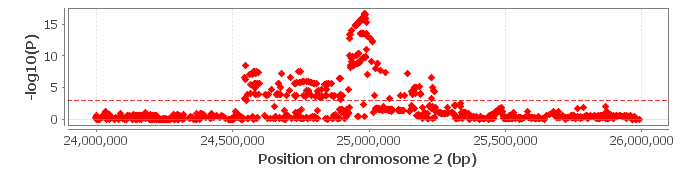


Figure S5. Regional plot for ADCY3 locus showing expression in lymphoblastoid cell lines from 856 healthy female twins of the MuTHER resource. Each diamond represents a SNP plotted y its position on the chromosome against its association (-log10 P). Plots were created using Genevar ([4](#_ENREF_4)).

Table S2. Results of analysis of expression in lymphoblastoid cell lines at the *ADCY3* locus using data from 856 healthy female twins of the MuTHER resource ([5](#_ENREF_5)). Results were created using Genevar ([4](#_ENREF_4)). Only the first 20 SNPs are shown.

| SNP ID | SNP Position | A1 | INFO | Freq1 | beta_SNP | sebeta_SNP | chi | P |
| --- | --- | --- | --- | --- | --- | --- | --- | --- |
| rs6733224 | 24984411 | T | 0.973 | 0.534 | 0.1172 | 0.0139 | 70.78 | 2.02E-17 |
| rs11689546 | 24983955 | G | 0.982 | 0.463 | -0.116 | 0.0139 | 69.74 | 3.43E-17 |
| rs10198275 | 24984046 | C | 0.982 | 0.463 | -0.116 | 0.0139 | 69.74 | 3.43E-17 |
| rs6545814 | 24984820 | G | 0.982 | 0.463 | -0.116 | 0.0139 | 69.74 | 3.43E-17 |
| rs10200566 | 24983966 | T | 0.982 | 0.537 | 0.1159 | 0.0139 | 69.69 | 3.52E-17 |
| rs6721750 | 24982223 | G | 0.996 | 0.456 | -0.1139 | 0.0138 | 67.966 | 8.44E-17 |
| rs6712981 | 24979734 | G | 0.995 | 0.455 | -0.1134 | 0.0138 | 67.356 | 1.15E-16 |
| rs6726199 | 24979832 | G | 0.995 | 0.545 | 0.1134 | 0.0138 | 67.354 | 1.15E-16 |
| rs6545809 | 24980219 | T | 0.995 | 0.455 | -0.1133 | 0.0138 | 67.35 | 1.15E-16 |
| rs6706316 | 24981855 | G | 0.995 | 0.455 | -0.1133 | 0.0138 | 67.344 | 1.16E-16 |
| rs6722587 | 24985696 | C | 0.995 | 0.545 | 0.1133 | 0.0138 | 67.341 | 1.16E-16 |
| rs6724772 | 24982234 | T | 0.994 | 0.545 | 0.1133 | 0.0138 | 67.338 | 1.16E-16 |
| rs11900505 | 24985490 | C | 0.995 | 0.455 | -0.1133 | 0.0138 | 67.338 | 1.16E-16 |
| rs3903070 | 24976967 | G | 0.997 | 0.455 | -0.1133 | 0.0138 | 67.233 | 1.22E-16 |
| rs4077678 | 24976344 | G | 0.996 | 0.455 | -0.1133 | 0.0138 | 67.178 | 1.26E-16 |
| rs6756609 | 24974629 | C | 0.998 | 0.545 | 0.1132 | 0.0138 | 67.136 | 1.29E-16 |
| rs6713978 | 24974355 | T | 0.998 | 0.545 | 0.1132 | 0.0138 | 67.131 | 1.29E-16 |
| rs6723803 | 24974217 | G | 0.998 | 0.545 | 0.1132 | 0.0138 | 67.13 | 1.29E-16 |
| rs6545800 | 24972389 | T | 0.998 | 0.455 | -0.1132 | 0.0138 | 67.129 | 1.29E-16 |
| rs6752483 | 24973590 | T | 0.997 | 0.455 | -0.1132 | 0.0138 | 67.103 | 1.31E-16 |

1. Li Y, Willer C, Sanna S, Abecasis G. Genotype imputation. Annual review of genomics and human genetics. 2009; 10: 387-406.

2. Li Y, Willer CJ, Ding J, Scheet P, Abecasis GR. MaCH: using sequence and genotype data to estimate haplotypes and unobserved genotypes. Genetic epidemiology. 2010; 34(8): 816-34.

3. Howie B, Fuchsberger C, Stephens M, Marchini J, Abecasis GR. Fast and accurate genotype imputation in genome-wide association studies through pre-phasing. Nature genetics. 2012; 44(8): 955-9.

4. Yang TP, Beazley C, Montgomery SB, Dimas AS, Gutierrez-Arcelus M, Stranger BE, et al. Genevar: a database and Java application for the analysis and visualization of SNP-gene associations in eQTL studies. Bioinformatics (Oxford, England). 2010; 26(19): 2474-6.

5. Grundberg E, Small KS, Hedman AK, Nica AC, Buil A, Keildson S, et al. Mapping cis- and trans-regulatory effects across multiple tissues in twins. Nature genetics. 2012; 44(10): 1084-9.
